# Multi‐Target Drug Design in Alzheimer's Disease Treatment: Emerging Technologies, Advantages, Challenges, and Limitations

**DOI:** 10.1002/prp2.70131

**Published:** 2025-06-18

**Authors:** Md Saad Hossain, Md Hamed Hussain

**Affiliations:** ^1^ Department of Genetic Engineering and Biotechnology East West University Dhaka Bangladesh

**Keywords:** artificial intelligence, blood–brain barrier, computational drug design, drug discovery, multi‐target therapy, neuroinflammation

## Abstract

Alzheimer's disease (AD) is a complex and multifactorial neurodegenerative disorder, recognized as the most prevalent form of dementia. It is characterized by multiple pathological processes, including amyloid‐beta accumulation, neurofibrillary tangles, and neuroinflammation. The therapeutic efficacy of traditional single‐target drugs has been limited, failing to cure, halt, or reverse disease progression. Therefore, this complex disease warrants comprehensive therapeutic strategies like multi‐target drug design (MTDD). MTDD represents a promising strategy to target multiple pathological pathways concurrently. The integration of advanced technologies, including artificial intelligence, machine learning, and nanomedicine, can further enhance the precision and effectiveness of MTDD. This review explores various MTDD approaches, including multi‐target‐directed ligands, multi‐target compound combinations, and polypharmacology. These strategies aim to address the multifaceted nature of AD pathology more effectively than single‐target approaches. MTDD offers key advantages, including pathway‐level synergy, broader therapeutic scope, and potential for improved efficacy. However, MTDD faces various challenges and limitations, such as the complexity of drug design, difficulty of crossing the blood–brain barrier, and regulatory hurdles. Despite these challenges, recent advancements in computational methods and drug delivery systems show promise in overcoming these barriers. Future research should focus on optimizing delivery systems, improving in silico modeling, and translating multi‐target strategies into clinically viable therapies for AD. This review addresses these needs by critically analyzing recent technologies, advantages, challenges, limitations, and future directions of MTDD, underscoring its potential to transform AD treatment.

**Abbreviations**
5‐HT: 5‐hydroxytryptamine
ACh: Acetylcholine
AChE: Acetylcholinesterase
AD: Alzheimer's disease
AI: Artificial intelligence
ALS: Amyotrophic lateral sclerosis
APOE: Apolipoprotein E
APP: Amyloid precursor protein
Aβ: Amyloid beta
BACE‐1: β secretase‐1
BBB: Blood–brain barrier
BuChE: Butyrylcholinesterase
CADD: Computer‐aided drug design
CDK‐5: Cyclin‐dependent kinase 5
CNN: Convolutional neural network
CNS: Central nervous system
CRBN: Cereblon
EGFR: Epidermal growth factor receptor
FBDD: Fragment‐based drug discovery
FDA: Food and Drug Administration
GSK‐3β: Glycogen synthase kinase 3 beta
GWAS: Genome‐wide association studies
HDAC: Histone deacetylase
HER2: Human epidermal growth factor receptor 2
LSTM: Long short‐term memory
MAO: Monoamine oxidase
ML: Machine learning
MLP: Multi‐layer perceptron
MPO: Multi‐parameter optimization
MTCC: Multi‐target compound combination
MTD: Multi‐target drug
MTDD: Multi‐target drug design
MTDL: Multi‐target‐directed ligand
NFT: Neurofibrillary tangle
NMDA: N‐methyl‐D‐aspartate
NO: Nitric oxide
PD: Parkinson's disease
PLD3: Phospholipase D3
PSEN1: Presenilin 1
PSEN2: Presenilin 2
q‐RASAAR: Quantitative read‐across structure–activity–activity relationship
q‐RASAR: Quantitative read‐across structure–activity relationship
RNAi: RNA interference
ROS: Reactive oxygen species
siRNA: Small interfering RNA
SMILES: Simplified molecular‐input line entry system
SNALP: Stable nucleic acid lipid particle
SVM: Support vector machine
TCnet: Target Combination Network
TCscore: Target Combination Score
VHL: Von Hippel–Lindau

## 1 Introduction

### 1.1 Background of AD

Alzheimer's disease (AD) is the most common form of dementia among neurodegenerative diseases, characterized by loss of memory, thinking, reasoning, and behavioral changes in the elderly population. It is a gradually advancing condition that induces pathological alterations in the brain long before the manifestation of clinical symptoms [1, 2]. The World AD 2018 report mentioned that a new dementia patient is born every 3 s around the world. In 2006, there were 26.6 million people globally affected by AD. Projections suggest that by 2050, this number could increase to approximately 106.8 million worldwide. Within Europe, it is estimated that there will be around 16.51 million patients [3, 4, 5]. Additionally, the population of Americans aged 65 and older is expected to grow from 55 million in 2020 to 94 million by 2060 [6], further contributing to the disease burden.

**FIGURE 1:**
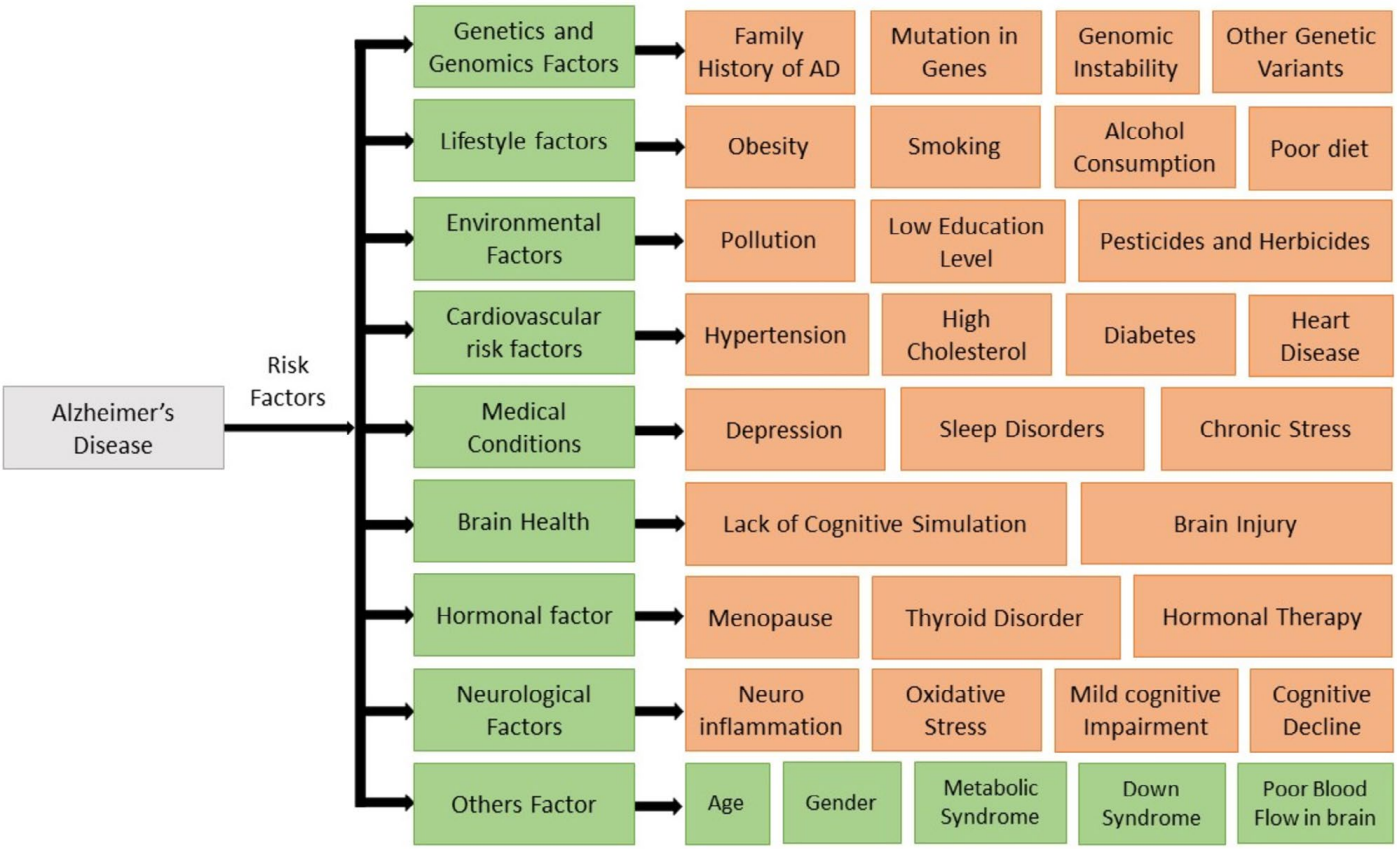
Overview of risk factors associated with AD. This schematic categorizes the multifactorial risk factors contributing to AD into primary domains (highlighted in green) and additional influences (highlighted in orange). Each main category represents a key factor, while the sub‐factors detail specific contributing elements.

The exact cause of AD remains undiscovered. Initially, AD was considered a rare condition and later thought to be a normal part of the aging process. Social stigma and various other factors historically impeded proactive research, therapeutic considerations, and treatment for AD patients [7]. However, these misconceptions are gradually diminishing. Though the exact etiology of AD remains unknown, various risk factors have been indicated including age, gender, lower educational and occupational attainment, prior head injuries, sleep disorders, cholesterol, epigenetic factors, down syndrome, estrogen replacement therapy, cardiovascular risk, hypertension, and many more [8, 9, 10, 11, 12, 13]. The complex nature of risk factors for AD is schematically represented in Figure 1. While initial treatments showed only modest efficacy, there is a growing array of available interventions. Yet, no drug exists that can fully cure AD, making the search for an effective disease‐curing treatment a challenging task. This leaves scientists with the difficult job of understanding the highly intricate mechanisms of neurodegeneration [14, 15].

AD is a slowly progressing and irreversible neurological illness that ultimately leads to the destruction and death of brain cells. The hallmark pathological features of AD include accumulation of amyloid‐beta (Aβ) plaques, neurofibrillary tangles (NFTs) composed of hyperphosphorylated tau protein, decrease of the neurotransmitter acetylcholine (ACh), neuroinflammation of the central nervous system, metabolic homeostasis disruption of metals, oxidative stress, β secretase‐1 (BACE‐1) activation, and widespread neuronal loss [16, 17]. Despite significant advances in understanding the pathology of the disease, effective treatment has remained elusive. AD progresses from very mild cognitive impairment to severe dementia, and its prevalence is expected to rise with the aging population. From 2002 to 2012, only one out of 244 tested compounds for AD has gained FDA approval, corresponding to a success rate of merely 0.4% [18]. This low success rate is widely attributed to the predominance of single‐target therapeutic strategies during that period. Notably, recent years have witnessed a promising shift in the AD drug development landscape. From 2014 to 2024, the number of investigational agents has increased by 96.8%, with 187 compounds currently undergoing clinical evaluation. This trend reflects a growing emphasis on disease‐modifying approaches, moving beyond traditional symptomatic treatments [19]. Given the limited efficacy of current FDA‐approved drugs, which offer only temporary symptomatic relief by targeting single pathways such as acetylcholinesterase (AChE) inhibition or N‐methyl‐D‐aspartate (NMDA) receptor modulation, there is a pressing need for innovative approaches [20]. In this context, multi‐target‐directed drug design (MTDD) has emerged as a promising strategy. By concurrently modulating multiple pathological pathways involved in AD, MTDD holds potential to overcome the limitations of traditional monotherapies and provide more comprehensive and effective treatment options [21, 22].

### 1.2 Significance of MTDD

AD results from an interplay between genetic, molecular, and environmental factors. Dysregulation across various biological processes, including enzyme functioning, protein–protein interactions, balance between neurotransmitters, oxidative stress, homeostasis of metal ions, and protein misfolding, underlies its pathogenesis [23, 24, 25]. This complex network poses a significant challenge for efficacious treatment, especially for traditional single‐target medicines that do not fully treat the disease's wide range of pathological characteristics. The complexity of AD pathology involves multiple molecular targets and pathways, making drug development a challenging task. Traditional single‐target therapies are largely unable to deliver comprehensive benefits because they do not sufficiently address the multifaceted nature of this disease. Conversely, MTDD holds promise for developing compounds that can simultaneously modulate multiple pathogenic factors, offering a more effective and holistic approach to treatment [26, 27, 28]. The multifactorial nature of AD supports the theory that successful therapeutic treatment for this type of disorder requires MTDD.

Multi‐target‐directed ligands (MTDLs) are compounds designed to concurrently modulate the processes of Aβ aggregation, tau phosphorylation, generation of oxidative stress, inflammation, and synaptic dysfunction, all of which are biological targets associated with AD pathogenesis. By targeting several pathways concurrently, MTDLs may exert synergistic therapeutic effects, enhance efficacy, and reduce the risk of drug resistance. The shift toward the MTDL strategy represents a novel approach in the development of anti‐AD therapies [29, 30, 31]. In this context, in silico drug discovery, utilizing advanced computational methods has emerged as a powerful tool for identifying and optimizing novel drug candidates [32]. Concurrently, new drug development strategies focus on multi‐target compound combinations (MTCCs) by integrating several pharmacophores into a single medication through molecular hybridization techniques, such as conjugated, fused, or merged scaffolds [33, 34, 35]. These methods, which speed up the identification, screening, and lead optimization of novel drugs, are powered by computational drug discovery techniques like machine learning (ML), deep learning, and computer‐aided drug design (CADD). These technologies offer an efficient platform for rapid compound screening and optimization, facilitating faster drug development and improving the potential for effective multi‐target therapies [36, 37].

For a detailed integration of such developments, literature from published, peer‐reviewed studies sourced from databases such as PubMed, Google Scholar, and ScienceDirect, with terms such as “Alzheimer's disease,” “multi‐target drug design,” “polypharmacology,” and “emerging technologies in AD” has been utilized. Notably, more than 95% of the cited literature is from 2019 to 2025, reflecting an emphasis on the latest and most impactful advancements in this field. Building on this extensive literary foundation, this review provides a comprehensive overview of modern MTDD for AD therapy, including the rationale behind multi‐targeting approaches, their development, and relevant studies on currently available multi‐target drugs (MTDs). It also discusses the advantages, challenges, and limitations of MTDD, such as therapeutic coverage, side effects, drug development complexity, clinical trial challenges, and regulatory hurdles. By critically examining these aspects and bridging the fields of computational biology, medicinal chemistry, and AD research, this study aims to identify the potential impacts of multi‐targeting drugs on AD treatment paradigms and highlight future research needs in this area.

## 2 Pathophysiology and Key Molecular Targets in AD

### 2.1 Types of AD

AD can be divided into two subtypes. The first type of AD, known as “early‐onset” AD (EOAD), is linked to genetic changes, particularly those affecting presenilin 1 (PSEN1), presenilin 2 (PSEN2), and amyloid precursor protein (APP) genes, which are involved in the synthesis of Aβ peptides. The second type of AD is known as late‐onset AD (LOAD), which is the most common type and generally affects people older than 65 years. It has been repeatedly linked to the apolipoprotein E (APOE) gene [38, 39]. Approximately 95% of AD patients suffer from LOAD, a complex disorder in which environmental factors and genetic predisposition both contribute to the pathology. The primary genetic risk factor for LOAD is the four APOE gene alleles [40]. Apart from APOE, several broadly distributed single nucleotide variants linked to lowered illness risk have been discovered by comprehensive genome‐wide association studies (GWASs) and the ensuing meta‐analyses. EOAD can be caused by mutations in the APP, PSEN1, or PSEN2 genes, which account for less than 5% of AD cases [41, 42, 43].

### 2.2 Complexity and Multifactorial Nature of AD

**FIGURE 2:**
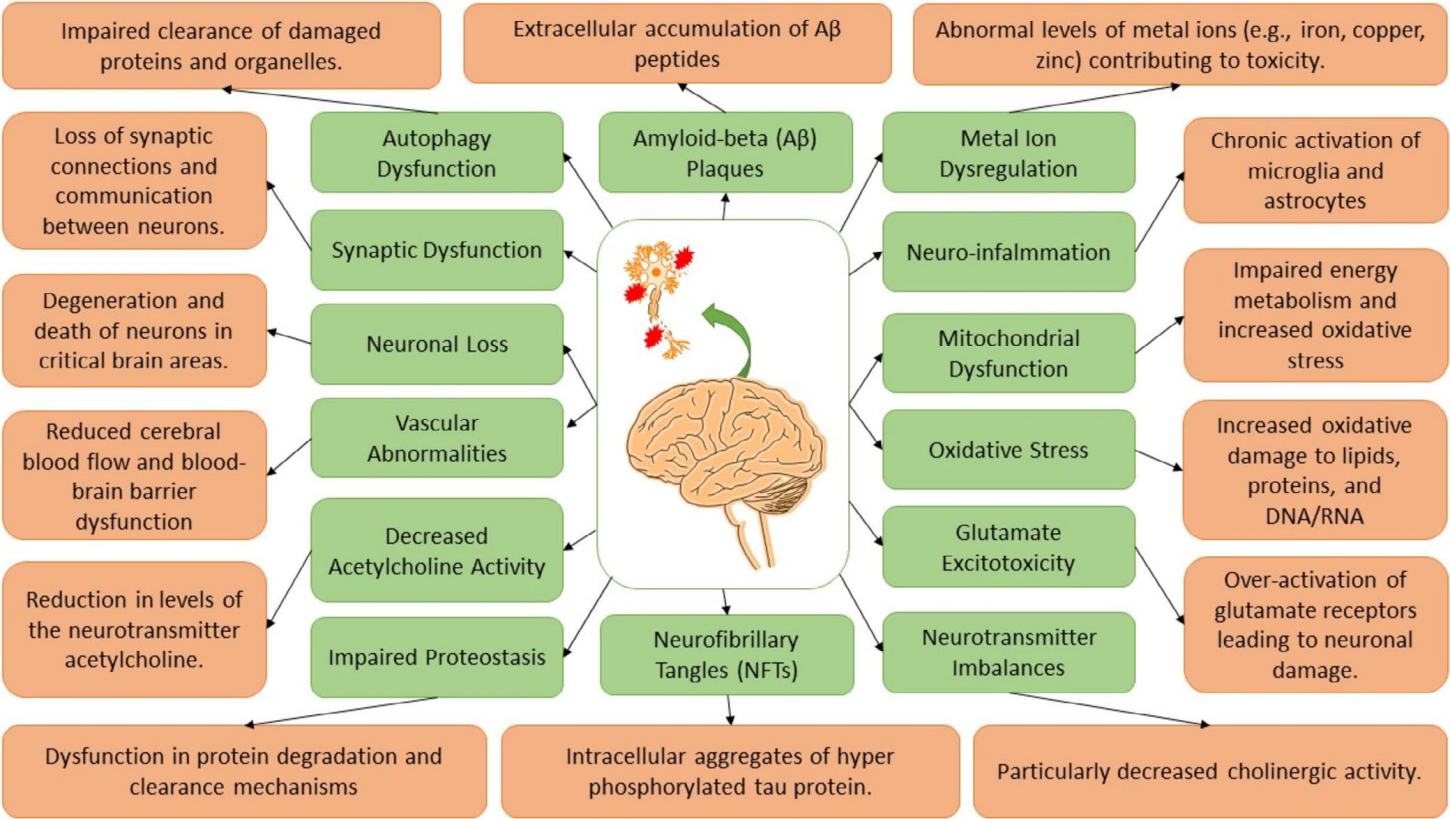
Hallmarks of AD and their pathological consequences. This diagram maps the core molecular and cellular hallmarks of AD (highlighted in green) alongside their respective pathological processes (highlighted in orange). It visually illustrates the progression from initial molecular disruptions to broader cellular and tissue‐level consequences.

Histopathological studies have revealed the multifaceted nature of AD, leading to various hypotheses about the disease's potential causes and effective targets. Significant research indicates that the development of NFTs and the deposition of amyloid plaques are the two main pathological features of AD [44, 45, 46]. AD is typically characterized by the presence of extracellular Aβ plaques, intracellular NFTs, brain atrophy, ventricular enlargement, and cortical and limbic system degeneration. Four major theories are widely recognized in explaining the pathogenesis of AD. The first posits that β‐amyloid protein aggregation in the brain induces oxidative stress, inflammation, and apoptosis. The second theory centers on the formation of intracellular NFTs due to hyperphosphorylated tau protein. The third is the cholinergic hypothesis, which suggests that a decline in ACh levels contributes to cognitive impairment. The fourth theory involves excitotoxicity triggered by NMDA receptor overactivation [39, 47, 48]. The cholinergic hypothesis, amyloid cascade hypothesis, tau hypothesis, mitochondrial cascade hypothesis, oxidative stress hypothesis, excitotoxicity hypothesis, neuro‐inflammatory hypothesis, and other theories involving genetic and environmental factors are some of the numerous hypotheses that have emerged in relation to AD [49, 50]. Additionally, intracellular hallmarks of AD include genomic instability, macromolecular damage, protein damage, lipid damage, and epigenetic alteration [51]. Figure 2 illustrates different hallmarks and their pathological processes contributing to the onset and progression of AD.

**FIGURE 3:**
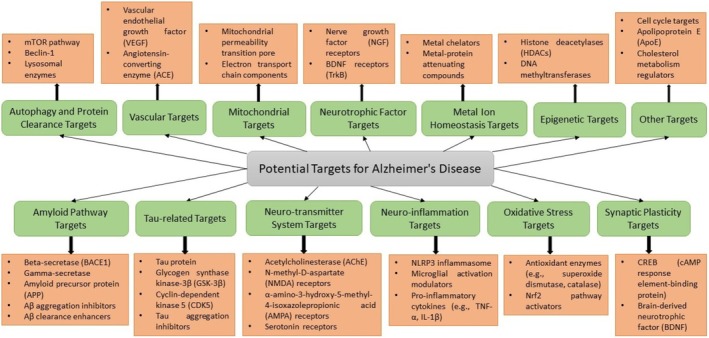
Therapeutic targets and molecular pathways involved in AD pathogenesis. This schematic highlights key therapeutic targets in AD's major pathological domains (highlighted in green) and their associated molecular pathways or components (highlighted in orange). It emphasizes the interconnected nature of AD's pathophysiology and illustrates the rationale for multi‐target drug design strategies aimed at simultaneously addressing multiple pathological processes.

Understanding AD's key pathological mechanisms and their associated molecular targets is important for the development of effective drugs [52]. Among the key targets associated with AD are AChE, β‐site APP cleaving enzyme‐1, glycogen synthase kinase 3 beta (GSK‐3β), monoamine oxidases (MAOs), metal ions in the brain, NMDA receptor, 5‐hydroxytryptamine (5‐HT) receptors, the third subtype of histamine receptor, and phosphodiesterases [26, 53]. In Figure 3, various therapeutic targets and pathways involved in AD pathogenesis are shown. In the following sections, we will discuss some hallmarks of AD more closely, starting with the amyloid pathway and Aβ accumulation, which are considered primary features of AD pathology.

### 2.3 Amyloid Pathway and Aβ Accumulation

One of the primary pathological features of AD is the accumulation of Aβ plaques. The amyloid pathway initiates with the abnormal processing of APP by BACE‐1 and γ‐secretase, which leads to excessive production of Aβ peptides. The enzymatic activity leads to the overproduction of the Aβ peptides, which eventually aggregate to form oligomers and plaques that subsequently cause synaptic dysfunction with concomitant induction of inflammation. The primary molecular targets of this pathway are APP, BACE‐1, γ‐secretase, proteins related to Aβ aggregation, and clearance [54, 55]. Mass spectrometric assays for plasma Aβ42 or Aβ42/40 have demonstrated high accuracy in detecting cerebral Aβ pathology [56]. Moreover, neuroinflammation, involving microglia, astrocytes, neurons, and oligodendrocytes, significantly influences the pathophysiology of AD. Although neuroinflammation can intensify brain damage, certain immune cells also play a role in Aβ clearance, brain repair, and regeneration. Understanding the dual role of immune cells in AD pathogenesis is essential for developing targeted interventions [57, 58].

### 2.4 Tau Pathology and NFTs

Tau pathology is the second most important hallmark of AD. This appears from hyperphosphorylation of the tau protein, which results in dissociation of the tau protein from the microtubules and subsequent aggregation into NFTs. The most important molecular targets are the tau protein itself and associated kinases responsible for tau phosphorylation, such as GSK‐3β and cyclin‐dependent kinase 5 [59, 60]. AD is characterized by the spread of tau pathology throughout the cerebral cortex, showing substantial variability in the population. Different subtypes of AD present with distinct demographic and cognitive profiles, implying a systematic variation in tau pathology that may affect treatment response and cognitive decline rates [61]. Mutations in the tau gene are linked to various neurodegenerative disorders, termed tauopathies, characterized by aggregated tau deposits.

Therapies targeting tau have shown promise, particularly through immunotherapy in mouse models. Inhibiting tau kinases to prevent tau phosphorylation regardless of the cause could be a potential therapeutic approach. Recent advances in tau positron emission tomography imaging, like the tracer T807, are aiding in understanding the distribution of tau pathology in AD and tauopathies [62, 63, 64]. Additionally, Vogel et al. revealed that pathological tau proteins can spread through synaptic connections, worsening neurodegenerative processes and leading to widespread brain atrophy. These findings underscore the critical need for therapeutic strategies targeting tau pathology [65].

### 2.5 Neuroinflammation of AD

Chronic neuroinflammation is another important factor responsible for the occurrence and progression of AD. Activated microglia and astrocytes release pro‐inflammatory cytokines and chemokines, which worsen Aβ and tau pathology while contributing to neuronal damage [66]. Among the significant molecular targets, the NLRP3 inflammasome has emerged as a key mediator of inflammatory responses in the last decade [67]. Studies have shown that astrocytes undergo morphological and functional alterations in AD, which can impact neuronal health and contribute to disease progression. Recent genetic studies have identified risk polymorphisms associated with AD, shedding light on the complex interplay between genetic factors and astrocytic function during disease pathogenesis. For instance, variations in genes such as CD33, ZCWPW1, CELF1, FERMT2, and INPP5D have been linked to AD risk and are known to regulate gene expression and modulate APP metabolism in astrocytes [68].

### 2.6 Oxidative Stress and Mitochondrial Dysfunction

Oxidative stress and mitochondrial dysfunction are intimately linked with AD pathogenesis. The high oxygen utilization of the brain combined with relatively weak defenses against antioxidants renders it highly vulnerable to oxidative injury [69]. Oxidative stress arises from a disequilibrium state in Reactive Oxygen Species (ROS) generation and the antioxidant defenses within the brain. ROS cause proteins, lipids, and DNA damage and further impair neuronal function and viability. Such oxidative damage not only contributes to but also exacerbates other pathological features of AD [70, 71]. On the other hand, the Mitochondrial Cascade Hypothesis proposes that age‐related changes in mitochondrial function play a critical role in the development of AD. Studies have shown that toxins‐induced mitochondrial dysfunction can drive Aβ production, which shows a link between mitochondrial dysfunction and Aβ accumulation in AD brains. Furthermore, mitochondrial DNA inheritance, particularly from the maternal lineage, has been implicated in AD risk, highlighting the role of genetic factors in mitochondrial function and disease progression [72, 73].

### 2.7 Synaptic Dysfunction and Vascular Abnormalities

Synaptic dysfunction and loss are initial events in AD that strongly correlate with cognitive decline. Impairment of the neurotransmitter system, specifically the cholinergic system, dramatically leads to memory and cognitive deficits. Therefore, synaptic proteins and neurotransmitter receptors can be viable targets for preserving normal mental processes and cognitive function [70, 74]. The segregation of functional networks in AD may enhance cognitive resilience [75, 76, 77]. The dysregulation of synaptic signaling and synaptic failure play a crucial role in the progression of AD, impacting neuronal communication and cognitive function. Mechanisms such as Aβ and tau protein have been implicated in synaptic toxicity, leading to structural and functional impairments at neuronal synapses.

Understanding the molecular and cellular mechanisms underlying synaptotoxicity is essential for developing targeted therapies to address synaptic dysfunction in AD [78]. In addition to synaptic issues, vascular abnormalities and dysfunction play a significant role in AD pathogenesis, encompassing alterations in cerebral blood flow and blood–brain barrier (BBB) integrity. Targeting molecules involved in vascular function and maintaining BBB integrity could be integral to a more comprehensive approach to AD treatment. The complex interplay between vascular health, innate immunity, and neuronal function, both independently and synergistically with amyloid and tau pathology, underscores the need to broaden our understanding of AD pathogenesis [79, 80].

### 2.8 Additional Pathways and Targets

In the realm of AD pathophysiology and potential therapeutic targets, recent research has highlighted the role of apolipoproteins as promising risk markers for AD. Specifically, investigations into the associations of various apolipoproteins, such as APOE and APOA‐I, with AD neuropathology have revealed intriguing insights [81]. These studies reveal that the dysregulation of metal ions, specifically copper, zinc, and iron, contributes to oxidative stress and Aβ aggregation that are key processes in AD progression [82, 83]. Additionally, defective neurotrophic factor signaling, mainly involving brain‐derived neurotrophic factor and nerve growth factor, impairs neuronal survival and plasticity. Besides, autophagy impairment and defective protein clearance mechanisms lead to the build‐up of toxic protein aggregates [84]. These interconnected pathological processes underline the complexity of AD. Some key targets in the pathogenesis of AD include APP, BACE‐1, tau protein, and activation of microglial cells. APP is a precursor molecule of Aβ generated from it, and the APP gene is a host to mutations that contribute to enhanced production of Aβ and enhanced propensity for aggregation [85, 86]. Furthermore, recent studies have suggested that gut microbiota dysbiosis may play a significant role in AD pathophysiology. Imbalances in gut microbial populations can trigger neuroinflammation and promote amyloid plaque formation, thereby accelerating neuronal loss and cognitive decline [87, 88].

BACE‐1 is the enzyme responsible for the initial critical cleavage of APP in Aβ production, making it an important therapeutic target. This indicates the formation of NFTs via hyperphosphorylation of tau protein. Hence, targeting tau phosphorylation and aggregation could be a promising approach for intervention to prevent or decrease NFT formation [89]. In the brain tissue of AD patients, AChE is more abundant than butyrylcholinesterase (BuChE), leading to the degradation of ACh in critical regions such as the hippocampus and cerebral cortex. The use of AChE inhibitors, such as donepezil, represents a key therapeutic strategy for AD by enhancing cholinergic neurotransmission and temporarily alleviating cognitive and behavioral symptoms. However, the clinical efficacy of ChE‐Is in modifying disease courses remains a topic of debate, with inconsistent results from various trials [90].

The PI3K/AKT signaling pathway plays a crucial role in the regulation of various biological processes and signal transduction mechanisms. The activation of this pathway protects neurons against Aβ‐induced neurotoxicity. Moreover, alterations in the expression of anti‐apoptotic proteins such as Bcl‐2 and Bcl‐xL have been implicated in AD‐related neuronal cell death. The PI3K/AKT pathway regulates the expression of these mitochondrial membrane permeability proteins, thereby influencing cell survival and apoptosis [91]. In AD, chronic activation of microglia is a key factor underlying sustained inflammation and neuronal damage. Modulating microglial activity to reduce inflammation presents a potential therapeutic avenue. For instance, Aβ accumulation can trigger both tau hyperphosphorylation and responses leading to inflammation and oxidative stress, further contributing to disease progression [92, 93].

The importance of phospholipase D3 (PLD3) in the pathophysiology of AD has been highlighted by recent investigations. PLD3 has been linked to the regulation of endolysosomal biogenesis, an essential process for preserving neuronal health. According to studies by Yuan et al., PLD3 overexpression causes a build‐up of large endolysosomal vesicles, which in turn causes axonal spheroids to form surrounding amyloid plaques. Moreover, the data imply that amyloid‐β deposits and PLD3 accumulation could work in concert to cause endolysosomal abnormalities, which would increase the neurodegenerative processes that define AD [94]. The intricate interplay between various molecular pathways, including amyloid accumulation, tau hyperphosphorylation, neuroinflammation, oxidative stress, and synaptic dysfunction, suggests that addressing multiple targets simultaneously may yield more effective therapeutic outcomes. These findings have led to a paradigm shift in AD drug discovery. By targeting key molecular pathways through a combinatorial approach, MTD therapies offer a more comprehensive and potentially effective strategy for treating AD.

## 3 Current Treatment Landscape

AD treatment is still a major issue in modern medicine, with available treatments offering limited efficacy in managing this complex neurological disease. The current treatment landscape for AD remains focused primarily on symptomatic relief, which is still the major goal of AD treatment, rather than treating the underlying causes of the illness [95]. The main objective of AD treatment is to develop cognition and memory by targeting various neuronal factors involved in the progression of AD [96, 97]. While there is no cure for AD, many drugs can help enhance cognitive function, reduce clinical deterioration, and relieve symptoms like memory loss [98, 99, 100].

### 3.1 Existing Pharmacological Interventions

Despite this ongoing challenge, the FDA has approved several medications for the treatment of AD. However, none of these drugs, including cholinesterase inhibitors like galantamine, rivastigmine, and donepezil, can completely cure the condition. These medications are primarily used to treat mild to moderate stages of the disease, offering some relief in symptoms and temporarily slowing cognitive decline [101, 102, 103, 104, 105, 106]. The main pharmacological interventions of AD are cholinesterase inhibitors and NMDA receptor antagonists [107]. Cholinesterase inhibitors work by increasing ACh levels, a neurotransmitter crucial for memory and cognitive function, by inhibiting its breakdown [108]. On the other hand, for moderate to severe AD, NMDA receptor antagonist memantine is often prescribed, and it is sometimes combined with a cholinesterase inhibitor [109]. Memantine helps regulate glutamate activity, potentially reducing excitotoxicity and improving cognitive performance. While these medications may slow disease progression, their therapeutic effectiveness could be further enhanced through innovative delivery systems designed specifically to target the brain [110, 111]. In addition to these, the FDA has approved newer treatments like aducanumab, an anti‐amyloid antibody administered via intravenous infusion [112]. Lecanemab, another antibody recently granted accelerated approval by the FDA, also targets Aβ plaques. Both these antibodies are thought to remove aggregates and Aβ plaques [113]. However, their long‐term impact on disease modification remains a question. The FDA approval of Aduhelm marks an important milestone in AD treatment, focusing on the removal of Aβ as a surrogate biomarker for disease progression rather than ameliorating cognitive symptoms. But it also has some side effects [114].

The current treatment landscape for agitation and psychosis in AD involves the use of second‐generation antipsychotics, despite safety concerns and their modest efficacy. Existing drugs for AD, like cholinesterase inhibitors and memantine, are approved for treating cognitive deficits but lack significant disease‐modifying activity against behavioral and psychological symptoms of dementia. New antipsychotic drugs like brexpiprazole and pimavanserin have shown positive clinical evidence for treating agitation and psychosis in AD, offering improved efficacy and safety profiles compared with current options [115]. In a recent study by Lee and colleagues, brexpiprazole at doses of 2 or 3 mg demonstrated statistically significant improvement in agitation compared with placebo over 12 weeks. It is a randomized clinical trial, which suggests that brexpiprazole is a promising option for managing agitation in patients with AD. Additionally, the study reported that brexpiprazole was generally well tolerated in the vulnerable patient population [116].

The variety of pathogenic pathways linked to AD suggests that combination therapy may be more beneficial than monotherapy in improving disease outcomes and lessening the impact of behavioral and social issues on patients' daily lives. Therefore, researchers are recently trying to develop combination therapy for AD treatment [117, 118]. While several combinations of drugs have been experimented and in the phase of clinical trials, only the Donepezil‐Memantine combination has received FDA approval for managing moderate to severe AD [119, 120]. Though combination therapy could prove to be more effective than monotherapy, it also involves higher costs. Therefore, further advanced research on AD treatments is essential.

### 3.2 Limitations of Single‐Target Drugs and Recent Therapies

For several decades, AD treatment strategies have predominantly relied on single‐target therapies, a major limitation in the fight against this complex disorder. This limitation has led to a paradigm shift toward MTD discovery. Research indicates that therapies focusing exclusively on single targets have not produced significant clinical improvements, disease modification, or meaningful outcomes [121, 122]. The response to FDA‐approved drugs can vary widely among patients. Some may experience significant cognitive benefits, while others see only modest improvements or no response at all. For instance, the cognitive benefits of Donepezil are typically temporary [123, 124, 125, 126]. Recent studies further highlight the challenges in AD therapy. Valdes et al. found that using human iPSC‐derived neuron models for EOAD poses limitations in studying sporadic forms of the disease, hindering our ability to fully understand disease mechanisms and develop effective treatments [127]. Another study by Thunell et al. identified a lack of robust evidence for certain drug classes associated with AD risk. Notably, the evidence for several drug classes, such as beta‐blockers, MAOIs, PPIs, BZDs, and DMARDs, relied primarily on observational studies, which have limitations in establishing causal relationships. This underscores the need for more diverse and rigorous research strategies to advance AD treatment [128].

The effectiveness of these single‐target therapies is limited. While these medications may provide temporary cognitive benefits and slow down the rate of cognitive decline in some patients, they do not stop the progression of AD [129]. The single‐target strategy frequently results in deficient clinical benefits and can have many side effects that affect AD patients. Common adverse effects include dizziness, nausea, and diarrhea, which are especially difficult for older individuals to handle [130, 131, 132]. This transient cognitive improvement emphasizes the need for comprehensive treatments that target multiple pathways and address the complex pathology of AD [133, 134].

### 3.3 Challenges in AD Drug Development

The development of new AD treatments faces many challenges. Key obstacles include limited evidence of the particular mechanism and difficulty in pinpointing a primary target for therapeutic intervention. Additionally, the lengthy and costly clinical trial processes coupled with the challenge of identifying reliable biomarkers for early diagnosis and treatment monitoring further complicate progress [49, 135]. Another major hurdle is the development of effective drug delivery to the central nervous system. The BBB restricts the passage of therapeutic molecules to the brain, limiting the efficacy of potential treatments. Moreover, age‐related changes in neuronal membranes and receptors can impact drug efficacy. This highlights the importance of considering age‐related modifications in preclinical studies [136, 137, 138]. Furthermore, the progressive nature of AD requires stage‐specific research and treatment strategies, as therapies effective in the early stages may prove ineffective in later stages of the disease [49]. The high failure rate of clinical trials in AD is often due to lack of efficacy or adverse effects in late‐stage trials [139, 140]. These difficulties highlight the need for a more nuanced approach, such as multi‐target medication design, which can offer more thorough and efficient AD treatment strategies [141]. By simultaneously targeting multiple pathways involved in AD pathogenesis, MTDs have the potential to offer greater therapeutic benefits and improve clinical outcomes.

## 4 MTDD for AD

The drug discovery and drug design landscapes are evolving rapidly with a deeper understanding of disease pathophysiology and advances in computational methods. Conventional and traditional approaches to single targets are evolving toward more holistic and system‐based strategies [142, 143, 144]. These developments are now integrated into the concept of Next‐generation drug design (NGDD), with the aim of developing more effective and precise therapeutic agents. An important part of this evolution is MTDD, which focuses on the development of compounds that interact concomitantly with several biological targets. NGDD leverages cutting‐edge technologies and methodologies, including ML algorithms, artificial intelligence (AI), deep learning, CADD, systems biology, network pharmacology, high‐throughput screening, and phenotypic profiling [145, 146, 147]. These revolutionary technologies of the modern era have emphasized drug discovery with huge datasets in molecular structures, target interaction, biological activities, identification, and optimization of MTDs [148].

### 4.1 Rationale for Multi‐Target Approaches in AD

The potential of multi‐target approaches is not just theoretical. Over the last several years, the FDA has approved several MTDs for various complex diseases, such as lung cancer, breast cancer, and neurodegenerative disorders, thereby proving the viability and effectiveness of this strategy [149]. Table 1 presents examples of recently approved MTDs for different diseases. Given that a number of these MTDs have succeeded in treating other complex diseases, this provides a strong precedent for pursuing similar strategies in AD. For instance, ASS234 represents a molecule that combines the effects of donepezil and propargylamine PF9601N. ASS234 targets many enzymes, including AChE, butyrylcholinesterase, and MAOs A and B [150, 151]. Another MTD, donecopride, combines AChE inhibitory activity with its 5‐HT4 receptor agonist activity of RS67333. Donecopride was created by combining donepezil and RS67333 into a single molecule. Donecopride not only inhibits AChE but also crosses the BBB, has a low toxic profile, and has shown precognitive effects in vivo [152]. These are examples illustrating the potential of multi‐target approaches for the development of effective AD treatments.

**TABLE 1:** List of FDA‐approved MTDs for various diseases.

Approved year	Drugs name	Type	Disease name	Targets name	Drug bank accession number
2001	Imatinib	Small molecule	Leukemia, myelodysplastic, systemic mastocytosis, hypereosinophilic syndrome, dermatofibrosarcoma protuberans, and GIST	CSF1R, ABL, c‐KIT, FLT3, and PDGFR‐β	DB00619
2006	Sunitinib	Small molecule	Renal cell carcinoma (RCC) and imatinib‐resistant GIST	VEGFR, PDGFR, c‐KIT, FLT3	DB01268
2006	Dasatinib	Small molecule	Acute lymphoblastic leukemia or chronic myeloid leukemia	ABL, SRC, c‐KIT, EPHA2, and PDGFR‐β	DB01254
2007	Lapatinib	Small molecule	Breast cancer	EGFR, HER2	DB01259
2007	Sorafenib	Small molecule	Unresectable liver carcinoma, RCC, and differentiated thyroid carcinoma	RET, VEGFR2, c‐Kit PDGFR‐B. BRAF	DB00398
2009	Pazopanib	Small molecule	Advanced RCC and advanced soft tissue sarcoma	VEGFR‐1, VEGFR‐2, VEGFR‐3, PDGFR‐α, PDGFR‐β, FGFR‐1, FGFR‐3, KIT, ITK, LCK	DB06589
2011	Aflibercept	Biotech	Age‐related macular degeneration	VEGF‐A, VEGF‐B, PlGF	DB08885
2011	Crizotinib	Small molecule	NSCLC	ALK, ROS1, MET	DB08865
2011	Vandetanib	Small molecule	Medullary thyroid cancer	EGFR, VEGFR2, RET	DB05294
2012	Ponatinib	Small molecule	Chronic myeloid leukemia	ABL, c‐Kit	DB08901
2012	Tofacitinib	Small molecule	Rheumatoid arthritis, ankylosing spondylitis, and ulcerative colitis	JAK1, JAK2, JAK3	DB08895
2012	Bosutinib	Small molecule	Chronic myelogenous leukemia	Dual SRC, ABL Tyrosine kinase	DB06616
2012	Regorafenib	Small molecule	Metastatic colorectal cancer, metastatic GIST, and hepatocellular carcinoma	VEGFR2, c‐kit, TIE2, FGF, PDGF	DB08896
2013	Afatinib	Small molecule	Non‐small cell lung cancer (NSCLC)	EGFR, HER1, HER2	DB08916
2013	Dabrafenib	Small molecule	Melanoma, NSCLC, and thyroid cancer	BRAF, CRAF	DB08912
2014	Ceritinib	Small molecule	NSCLC	ALK, IGF‐1R, InsR, ROS1	DB09063
2014	Nintedanib	Small molecule	Idiopathic pulmonary fibrosis, systemic sclerosis‐associated interstitial lung disease, and in combination with docetaxel for NSCLC	VEGFR2, PDGFR‐a, PDGFR‐B	DB09079
2015	Alectinib	Small molecule	NSCLC	ALK, LTK, CHEK2, FLT3, PHKG2, and RET	DB11363
2015	Lenvatinib	Small molecule	Thyroid cancer, advanced RCC in combination with everolimus, and unresectable hepatocellular carcinoma	VEGFR1‐3, FGFR1‐4, PDGFR‐α, KIT, RET	DB09078
2015	Palbociclib	Small molecule	HER2‐negative and HR‐positive advanced or metastatic breast cancer	CDK4, CDK6	DB09073
2016	Cabozantinib	Small molecule	Advanced RCC, hepatocellular carcinoma, and medullary thyroid cancer	MET, VEGFR, RET, KIT, AXL	DB08875
2017	Midostaurin	Small molecule	Acute myeloid leukemia, aggressive systemic mastocytosis, and systemic mastocytosis	FLT3, KIT, PDGFR, VEGFR2, PKC	DB06595
2019	Fedratinib	Small molecule	Myelofibrosis	JAK2, FLT3, RET	DB12500
2019	Polivy (Polatuzumab vedotin)	Biotech (antibody‐drug conjugate)	large B‐cell lymphoma	CD79b, MMAE	DB12240
2019	Enhertu (trastuzumab deruxtecan)	Biotech (antibody‐drug conjugate)	HER2 positive breast cancer	HER2, Topoisomerase I	DB14962
2020	Selpercatinib	Small molecule	RET‐driven NSCLC, medullary thyroid cancer, and thyroid cancer	RET, VEGFR1‐3	DB15685
2020	Trodelvy (Sacituzumab govitecan)	Biotech (antibody‐drug conjugate)	Triple‐negative breast cancer	BCL‐2, CD20, Trop‐2	DB12893
2021	Belzutifan	Small molecule	Certain cancers associated with von Hippel–Lindau disease	HIF‐2α, EGFR, VEGFR2	DB15463
2022	Tabelecleucel	Biotech	EBV‐associated post‐transplant lymphoproliferative disorder	EBV‐specific T cells (multiple targets)	DB17072
2023	Elranatamab	Biotech	Multiple myeloma	BCMA, CD3	DB15395

Recent advances in rational design have produced multi‐target compounds with well‐defined biological properties and balanced affinities for multiple targets. Successful MTDD hinges on selecting the right combination of targets and optimizing in vivo profiles [149]. The increasing approval of MTDs by the FDA underscores the growing demand for such treatments. Lessons from successful MTD development in other diseases can guide AD drug discovery and development efforts.

### 4.2 MTDD: Strategies, Emerging Approaches, and Innovative Technologies

MTD discovery represents a radical paradigm shift in drug development. This strategy aims to design or identify compounds that can simultaneously modulate multiple disease‐relevant targets [153, 154]. Several key strategies have emerged, including MTDLs, MTCCs, and the concept of polypharmacology, along with other emerging approaches.

#### 4.2.1 Polypharmacology

The concept of polypharmacology has gained increasing attention in recent years, shifting from being viewed as a challenge in drug development to a potential advantage in MTD discovery. Since a single drug can modulate multiple targets of a pathway linked to the progression of a complex disease and exert therapeutic effects, polypharmacology has received considerable attention in the field of drug discovery in recent years [155]. Recent advances in drug development favor multitargeted polypharmacological strategies, exemplified by multifunctional pharmaceuticals that can be identified through drug repurposing and rational design [156]. Polypharmacology has the ability to target many pathogenic processes in AD. By considering the complex interactions among drugs, targets, and biological pathways, polypharmacology aligns well with the complexity of AD. This opens up new possibilities for drug repurposing and the identification of novel therapeutic targets through unexpected drug‐target interactions. Polypharmacology aligns well with the complexity of AD, where multiple interconnected pathways drive disease progression [157, 158].

#### 4.2.2 Multi‐Target‐Directed Ligands

MTDs are compounds designed to affect numerous disease‐relevant targets in order to improve safety and efficacy. MTDLs are an important aspect of MTDD, as they provide single molecules capable of interacting with many pathogenic processes in AD [159, 160]. For example, the conjugation of rivastigmine (RIV), a well‐known AChE inhibitor, with hydroxyphenyl benzimidazole (BIM) moieties has been reported as a multi‐target strategy. RIV–BIM hybrids combine cholinesterase inhibition, metal ion binding, Aβ aggregation inhibition, and antioxidant activity to target various facets of AD [35]. Lembo et al. found that dual‐target MTDLs can effectively control AD‐related targets, including drugs that inhibit AChE and interact with Aβ, increasing cognitive function and lowering amyloid load [160]. This multi‐action strategy is particularly helpful when a single therapeutic target is not sufficient to treat a disease like AD.

**FIGURE 4:**
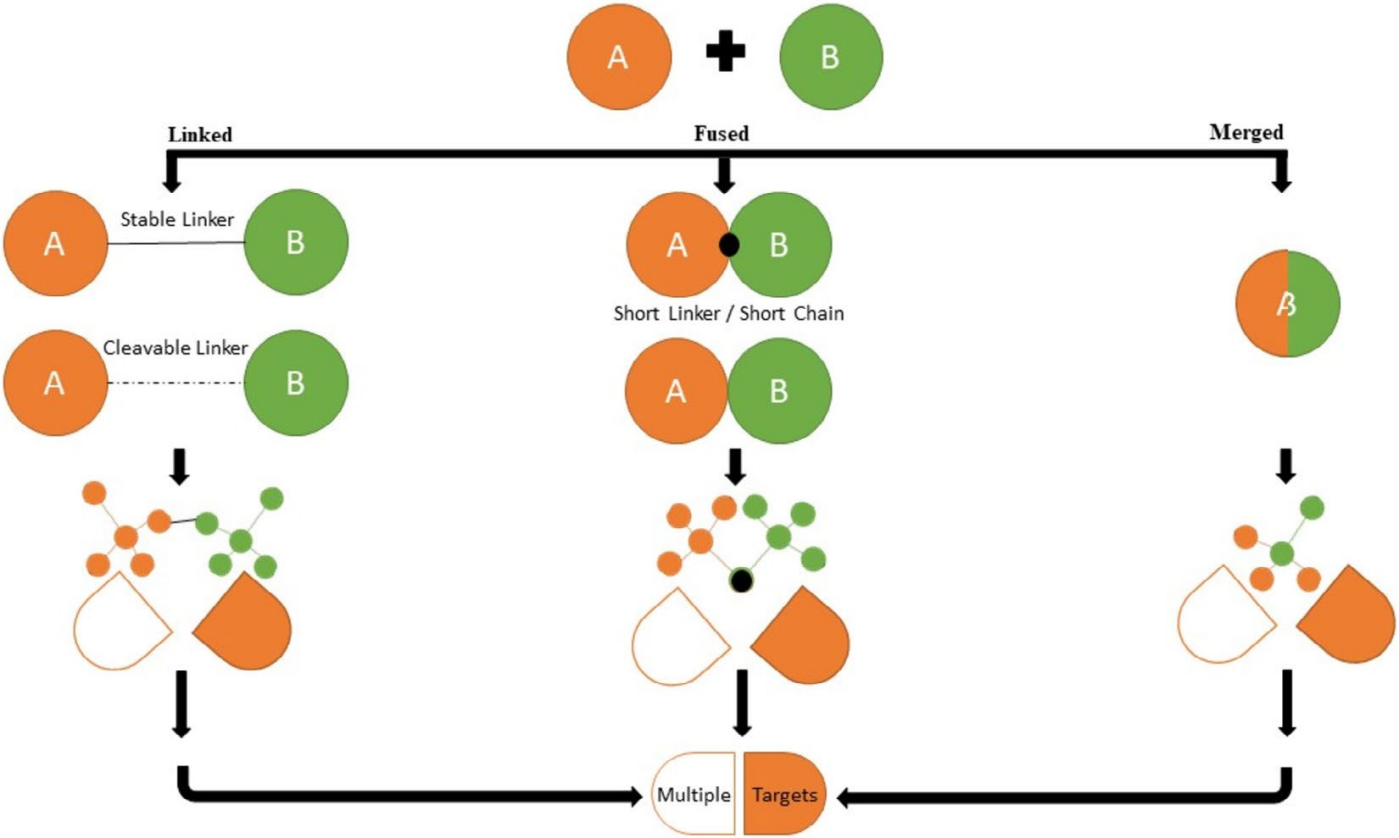
Design strategies for MTDLs: Linked, Fused, and Merged approaches. This schematic illustrates three fundamental approaches for designing MTDLs that interact with multiple biological targets. In the Linked approach, two distinct pharmacophores (A and B) are joined by a stable or cleavable linker. The Fused approach connects A and B with a short linker, achieving a closer integration while maintaining individual functionalities. The Merged approach combines overlapping structural elements from A and B into a novel hybrid pharmacophore.

The design of MTDLs leverages existing chemical scaffolds and employs computational tools like molecular docking, ADMET analysis, and molecular dynamics simulations to predict drug‐target interactions and optimize candidates efficiently [161, 162]. Design strategies include linked, fused, and merged approaches that combine pharmacophores targeting multiple disease pathways [159, 163, 164] In Figure 4, the three MTDL strategies are schematically represented. These three possible methods for producing a designed multi‐target ligand molecule using a “Framework Combination Approach,” exemplify how two compounds can be effectively combined. MTDLs developed through structure‐based drug design methods have demonstrated remarkable success, largely due to the growing accessibility of structural data for crucial targets in AD [159, 165].

#### 4.2.3 Multi‐Target Compound Combinations

MTCCs is another innovative approach in MTDD. Unlike MTDL, which is a single molecular entity, MTCC is carefully designed by combining two or more compounds together. These compounds incorporate more than one active ingredient, each targeted against a different aspect of AD pathology [166, 167]. This is a more flexible approach to the modulation of multiple targets because the individual components of a combination can be optimized independently. The rationale behind MTCCs is to achieve a synergistic effect of simultaneous actions on many pathological processes at the same time, which presents much more efficacy than single‐compound approaches [168, 169, 170]. Recently, new combinations of donepezil, memantine, and curcumin have been explored as a promising MTD in AD. Targeting the deficits in cholinergic systems, glutamatergic dysregulation, and neuroinflammation, this combination might lead to disease progression delay with an improvement in cognitive function [171]. A study by Yaghmaei et al. demonstrated that the combined use of Donepezil and Memantine significantly increased the probability of 5‐year survival among AD patients. This study was supported by robust data analysis from a large electronic health record dataset and highlights the potential benefits of favorable drug–drug interaction [172]. The development of MTCCs requires further research to understand drug–drug interactions, optimal dosing strategies, and the pharmacokinetics of individual components to ensure that the combined therapy is safe and effective. To better understand their strategies, a comparative overview of the key MTDD strategies is presented in Table 2. In this table, their distinct design approaches, various features, and applications in AD treatment are shown.

**TABLE 2:** Comparative summary of polypharmacology, multi‐target‐directed ligands (MTDLs), and multi‐target compound combinations (MTCCs) in AD therapy.

Feature	Polypharmacology	MTDLs (multi‐target‐directed ligands)	MTCCs (multi‐target compound combinations)
Definition	A drug's ability to interact with multiple targets, either within a pathway or across distinct biological processes	A single chemical entity intentionally designed to interact with two or more disease‐relevant targets for therapeutic synergy	A combination of two or more compounds, each targeting different aspects of the same disease
Design approach	Typically discovered through drug repurposing, omics profiling, or screening for off‐target activity	Designed via structure‐based methods, often using linked or merged pharmacophores	Involves empirical pairing or data‐driven selection of agents already known to act on complementary targets
Example	Repurposed CNS drugs with multi‐target effects in AD	RIV–BIM hybrids targeting AChE inhibition, Aβ aggregation, and ROS scavenging	Donepezil + Memantine and Donepezil + Curcumin combinations shown to improve cognitive function and survival in AD
Advantages	May offer enhanced efficacy, lower dose requirements, and broader therapeutic impact	Allows precise control of multi‐target activity within a single molecule	Offers flexibility in dosing, synergistic effects, and easier customization to patient‐specific pathologies
Challenges	Risk of off‐target effects, unclear dose–response behavior, and challenges in determining mechanisms of action	Requires complex synthesis and careful optimization of multi‐target binding	Higher chance of drug–drug interactions, pharmacokinetic mismatch, and the need for well‐controlled combination trials

#### 4.2.4 Emerging Innovative Technologies

In the context of MTDD, several technologies such as AI, RNA‐based therapeutics, PROTACs, multi‐omics integration, and quantum computing emerged to explore the interconnected pathways of AD. These approaches enable highly focused, multifaceted drug discovery beyond the scope of traditional methods. AI and ML‐based platforms can rapidly design effective MTDs by analyzing large datasets of molecular structures, target interactions, and biological activities [173]. The integration of AI and ML into MTD discovery creates another frontier in MTDD [174, 175, 176]. For example, Cerveira et al. observed that both Long Short‐Term Memory (LSTM) based and graph‐based genetic models can produce drug‐like molecules satisfying CNS permeability and multi‐parameter optimization (MPO) scores that could be directly utilized for the AD drug development [177].

Another study by Kongala et al. demonstrated the use of support vector machines (SVM) and multi‐layer perceptrons (MLP) on 3D MRI data to classify and predict AD with significantly higher accuracy (97.47%) and precision (97.42%) than traditional CNN models, highlighting the potential of integrating imaging with ML for MTDD validation and stratification [178]. Similarly, deep learning‐based diagnostic models have significantly contributed to Alzheimer's research, particularly in accurate disease classification and progression tracking. A convolutional neural network (CNN)‐based CAD system was developed using 18FDG‐PET images. The model achieved an accuracy of 96.8% [179]. Further advancements include a deep learning framework by Tsuji et al., which utilized protein–protein interaction networks and XGBoost models to predict novel drug‐target genes for AD. The DRIAD approach connects drug‐induced gene expression changes in neural cells to Braak stage progression, identifying compounds that may alleviate AD pathology [36]. Additionally, multi‐omics‐based ML platforms namely DrugCell have demonstrated potential for correlating omics signals with drug fingerprints to predict MTD efficacy [180]. Beyond predictive modeling, new technologies like deep generative architectures enable the direct creation of MTD candidates that can satisfy polypharmacological constraints. Mukaidaisi et al. generated over 1500 molecules by using a DEL‐based model which has 86.1% validity and 67.5% passing multi‐target binding thresholds for proteins [181].

Liu et al. suggested a MTDD model to design a molecule targeting protein sequences and feature similarity to steer molecule generation. Their model integrates a transformer‐based encoder with a generative gated recurrent unit‐based architecture to learn similarities between protein targets and generate SMILES strings with dual‐target binding, respectively. In molecular docking of the synthesized compounds against AD‐related targets, it revealed that the generated compounds had superior binding affinities of −12.9 and −8.5 kcal/mol for Aβ and tau, respectively, compared to conventional approaches [182]. Recent studies also investigated complex ML‐centered architectures such as q‐RASAR and q‐RASAAR models for AD's MTDD. Kumar et al. constructed single and dual‐target ML‐based models for seven most important AD targets (AChE, BuChE, BACE1, APP, Tau aggregation, CDK‐5, and 5‐HT6 receptors), showing strong predictive performance [183].

Multi‐omics integration enables concurrent analysis of transcriptomic, proteomic, and metabolomic data [184]. By using integrated transcriptomics and metabolomics, Liu et al. found that 3′,4′,5,7‐tetramethoxyflavone (TMF) significantly attenuated memory impairment. It can reduce Aβ plaques and tau phosphorylation. Also, it can suppress neuroinflammation in APP/PS1 mice models. These effects were associated with the alteration of the MAPK/NF‐κB signaling pathway and regulation of metabolic homeostasis with special emphasis on arginine and proline metabolism [185]. Zhou et al. combined multi‐omics and network analysis with Kai‐Xin‐San (KXS) formulation (a traditional Chinese medicine formulation) to uncover key metabolic and mitochondrial pathways in AD recovery [186]. Yue et al., integrated multi‐omics, and gene co‐expression networks to identify the most promising target combination by a multilayer random walk algorithm and developed TCnet. Their scoring system, TCscore, achieved a 100% recall rate in known MTD pairs and predicted combinations like GSK3β and ADAM17, which were later validated by molecular docking and cell assays. Among the traditional ingredients of HLJDT, quercetin was used as a leading component, which resulted in a 13.5% increase in cell viability and a 19.9% decrease in inflammation‐induced NO production. These findings demonstrate the importance of mechanism‐based target prediction enabled by network learning and integration of omics layers [187].

RNA interference (RNAi) strategies, in particular small interfering RNAs (siRNAs), have proven to be very promising for the design of MTDs for the treatment of neurodegenerative diseases like Alzheimer's. Multi‐target siRNA scaffolds are being engineered to simultaneously silence gene pairs implicated in AD pathogenesis, with strong in vivo efficacy and prolonged gene silencing in CNS tissues [188]. These types of dual‐target siRNAs have established comparable potency to conventional single‐target approaches. Although it offers simplified delivery and manufacturing advantages, many studies have shown the potential of structural modifications and delivery platforms like GalNAc conjugation and SNALP (Stable Nucleic Acid Lipid Particle) to enhance the stability and tissue‐specific accumulation of siRNA drugs. That leads to over 80% silencing of disease‐associated transcripts in preclinical models [189].

Drug repurposing in AD is a ML approach that evaluates the relationship between drug‐induced gene expression changes and the pathological stages of AD. This approach enables the unbiased interrogation of a biological process and drug candidates when the underpinning mechanism of the disease is not well understood. This is particularly useful because it often involves multiple co‐occurring pathologies [180]. Repurposing existing drugs known to have multi‐target effects is an attractive approach for AD because it leverages existing safety data and can expedite drug development. Li et al. identified 177‐gene amyloid‐β‐induced neuroinflammation modules by using co‐expression and spatial transcriptomics, linked it to specific microglia subtypes, and proposed 20 drug repurposing candidates based on network proximity [190].

Targeted protein degradation has been demonstrated to be an effective MTD discovery strategy for AD. PROTACs (Proteolysis‐Targeting Chimeras) form a promising approach in MTDD for AD via targeted degradation of AD‐related proteins such as tau. Many compounds that could decrease tau aggregates and recover synaptic function have been presented through CRBN and VHL‐based ligases. Furthermore, newer PROTAC designs with a peptide linker and the triazole ligation chemistry have shown better permeability across the BBB [191]. Zinc‐dependent histone deacetylases (HDACs), particularly HDAC6, are a promising epigenetic and multi‐target therapeutic entry point in AD. HDAC6 plays a dual role in modulating tau phosphorylation, influencing Aβ aggregation, and regulating protein clearance via autophagy and the ubiquitin‐proteasome system. Also, HDAC6‐targeted PROTACs are an advanced strategy by selectively degrading HDAC6 with a lower toxicity profile. It offers accurate control over protein levels, which is an essential feature in the context of complex diseases like AD [192].

Quantum computing is emerging as a game‐changer in CADD and MTDD. This has the potential to significantly reduce the time required for molecular simulations and docking. This advancement could lead to quicker identification of effective drug candidates for ad [193]. CADD uses computational algorithms to virtually screen millions of chemicals, selecting those with the greatest potential for multi‐target interactions. It also allows for the optimization of pharmacokinetic and pharmacodynamic features, enhancing drug safety and efficacy [194, 195]. Quantum computing simulates molecular interactions with atomic‐level precision. Many researchers applied quantum algorithms to analyze bond energy and interaction stability across candidate compounds. A study presented 92% inhibition and strong binding energy (−12.5 kcal/mol) for lead molecules. These models could compete with traditional approaches in terms of throughput and accuracy, which can pave the way for cost‐effective prioritization of MTDs in AD [196]. Finally, the integration of the cutting‐edge technologies represents a trend in the field of MTDs toward AD.

#### 4.2.5 Others Emerging Approaches

Emerging approaches in MTD discovery are further expanding the toolkit available to researchers. Network pharmacology, which uses systems biology to understand how drugs interact with multiple targets within biological networks, is providing new insights into the complex interplay of disease mechanisms [197, 198]. Fragment‐based drug discovery (FBDD) is a structure‐based strategy. Small, low‐molecular‐weight compound fragments are screened as part of FBDD to identify those that bind to the target protein. These compounds can be subsequently refined into powerful therapeutic leads. FBDD is not exclusively a multi‐target approach; it is being adapted to develop compounds that interact with multiple targets [199]. Phenotypic screening methods are also gaining popularity in recent times. These methods allow the identification of compounds with multi‐target effects based on their overall impact on cellular phenotypes rather than predefined molecular targets [200].

Nanomedicine is an emerging and promising approach for AD treatment that uses nanotechnology to enhance drug delivery and efficacy. Various nanocarriers like liposomes and electrospun nanofibers can be used for the encapsulation and targeted release of therapeutic agents to enhance their bioavailability and enable controlled release. This technology enables the simultaneous administration of multiple drugs [171]. Incorporating these various approaches into the MTD discovery paradigm holds great promise for the treatment of AD. Therefore, dealing with the multifaceted nature of AD through comprehensive therapeutic strategies may provide novel and more effective holistic treatment approaches that may slow down or even halt AD progression.

## 5 Advantages of MTDD for AD

MTDD represents a very significant advancement in the treatment of AD and has many advantages over traditional single‐target therapies. It requires a much more comprehensive treatment strategy because of the heterogeneous nature of AD, for which MTDs are highly suitable [160, 201]. Notably, recent data from FDA approvals suggest a growing success trajectory of this drug. From 2015 to 2017, 21% of newly approved small molecule drugs by the FDA were classified as MTDs. From 2000 to 2015, it was 16%. Also, the therapeutic combinations were about 10%. If we consider this part of the polypharmacological strategy, then the cumulative contribution rises to 31%. It becomes nearly equal to the share of single‐target drugs, which is about 34% approved in the same timeframe [202]. Several approved drugs demonstrate the success of multi‐target strategies in clinical practice. One example of successful MTD development is duvelisib. It is an oral dual inhibitor of PI3K‐δ and PI3K‐γ and it has shown high clinical efficacy in advanced hematologic malignancies. Another success of MTD is lapatinib, which targets both HER2 and EGFR tyrosine kinases. This drug has been approved for HER2‐positive breast cancer and offers a dual‐inhibition mechanism that improves treatment response compared to monotherapies. These two real‐world examples highlight how rationally designed MTDs can offer enhanced efficacy [203].

**FIGURE 5:**
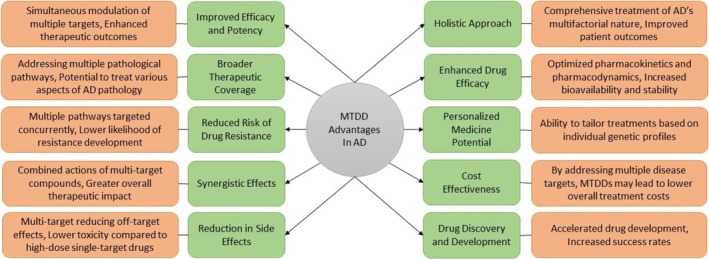
Advantages of MTDD in AD therapy. This diagram presents the key benefits of MTDD strategies in AD treatment. The central concept is encircled by 10 major advantages (highlighted in green), each supported by underlying mechanisms or therapeutic outcomes (highlighted in orange) that demonstrate the potential of MTDD approaches to address the complexity of AD pathology.

Over the last several years, the development of MTDs for AD has represented a significant shift from traditional single‐target strategies and offers numerous advantages. These advantages become even more important when many single‐target therapies have failed to significantly halt or reverse the progression of AD [184, 204]. An illustration of the advantages of MTDD in the treatment of AD is presented in Figure 5.

One of the primary benefits of MTDs is their potential for synergistic effects. It has the ability to target various interconnected mechanisms that contribute to disease progression. The synergistic effects will bring more treatment effects with an increase in effects on the slowing down of disease progression [204, 205, 206]. Additional advantages of multi‐target approaches include simplifying the treatment regimen, which can improve patient compliance and reduce the risk of drug–drug interactions typically associated with polypharmacy. Moreover, these strategies enhance the ability to penetrate the BBB while minimizing metabolism‐related toxicity issues [207, 208]. The BBB remains a significant obstacle in Alzheimer's treatment but a few MTDD strategies have shown success in drug delivery systems to overcome this challenge. For example, PROTAC‐based designs utilizing triazole ligation chemistry have demonstrated improved BBB permeability while simultaneously degrading key AD‐related proteins like tau through ligases such as CRBN and VHL. Additionally, studies on HDAC6‐targeted PROTACs revealed not only successful BBB penetration but also reduced toxicity due to selective degradation mechanisms [191, 192]. Another example is the development of the PROTAC compound XL01126 that is designed to degrade LRRK2 in Parkinson's disease, which showed successful oral bioavailability and BBB penetration in mouse models [209].

Also, RNA‐based therapeutics utilizing innovative delivery platforms have shown promise. GalNAc‐conjugated siRNA and SNALP systems have demonstrated high delivery efficiency across the BBB, achieving over 80% silencing of AD‐relevant transcripts in preclinical models. These platforms mainly enable multi‐target silencing of gene pairs involved in AD pathogenesis, and they also offer a delivery framework that combines molecular precision with BBB permeability [189]. However, traditional nanocarrier systems remain central to advancing MTDD. A recent study by Yuan et al. found receptor‐mediated transport and nanoparticle‐based carriers as pivotal innovations in targeting CNS diseases. The use of liposomes and functionalized nanoparticles with ligands for BBB transporters, for example, transferrin or insulin receptors, can enhance drug uptake into the brain. This integrative strategy of combining MTDD with nanotechnology provides a promising route for overcoming pharmacokinetic barriers while maintaining polypharmacological action [210].

Another key advantage of MTDD is that it could overcome the limitations related to the heterogeneity of AD. The disease presents differently among patients and with variable degrees of involvement of specific pathological processes. Thus, MTDD strategies can be designed to target multiple pathways related to the disease process, which may be relevant in individual patients and, thus, are potentially more personalized and effective treatment regimens [211]. Inhibiting many targets related to the process of disease simultaneously may decrease the probability of developing drug resistance that is common in single‐target therapies [212, 213]. This comprehensive approach may also reduce the individual doses of each component, the quantity of drug, cost, and toxicity, thereby reducing side effects and improving the overall safety profile of the treatment [214].

MTDs may also have a wider therapeutic window, thus reducing the likelihood of adverse effects at therapeutic doses. By distributing the drug's action across numerous targets, the risk of overwhelming a single pathway is minimized, potentially resulting in an overall more balanced and tolerable treatment profile [202, 215]. A fact that is especially important in AD is that most elderly patients are susceptible to negative adverse effects from medications. That is why, MTDD can be combined with other advanced drug discovery technologies, such as AI, ML, and high‐throughput screening, which would make its potential benefit far more significant [36, 216]. The success of MTDs in AD will indeed pave the way for innovative strategies for drug development against a broad spectrum of complex diseases. The ideas and approaches to designing MTDs for AD can be easily transformed into the research of other neurodegenerative diseases, such as Parkinson's disease, Huntington's disease, and Amyotrophic lateral sclerosis.

## 6 Challenges and Limitations of MTDD for AD

Not all MTD development efforts have been successful. Some MTDs face challenges, particularly in the clinical translation phase. Many anticholinergic drugs designed for AD have only shown marginal and transient effects that lack curative benefits and offer limited restraint on neurodegeneration. Even with significant pharmaceutical investment, most drug candidates targeting the amyloid hypothesis or dopamine‐based strategies have failed to move beyond academic research [203]. Not only these, but some of the top‐predicted MTDD compounds failed to translate into meaningful in vivo efficacy. For example, when tested in a Drosophila model of RET‐driven cancer, ZINC98209221 and other lead compounds failed to significantly rescue fly survival [217]. These failures demonstrate that while MTDD is conceptually strong, it is practically and experimentally challenging.

**FIGURE 6:**
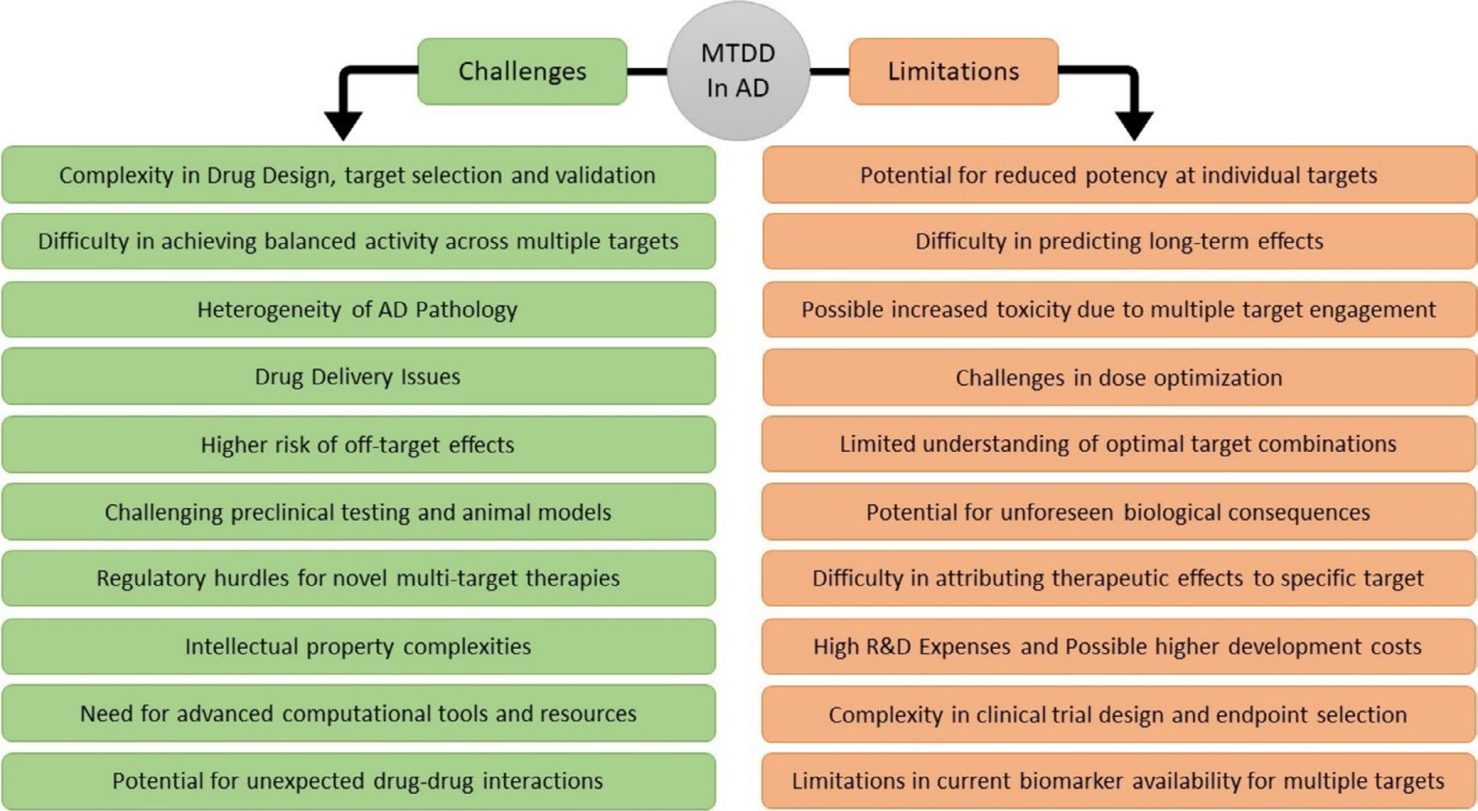
Challenges and limitations of MTDD in AD therapy. This figure presents a comprehensive overview of the practical and scientific challenges encountered in MTDD development for AD. The left panel (highlighted in green) outlines key challenges, while the right panel (highlighted in orange) outlines the inherent limitations of current approaches.

Drug design complexity stands as a major difficulty in AD. These challenges extend from the conceptual drug design stage to the clinical implementation and regulatory approval stage. As shown in Figure 6, the challenges and limitations of MTDD in AD are diverse and interconnected to each other. One of the primary hurdles is balancing the drug design process to ensure appropriate target selection and validation. Designing molecules that can modulate multiple pathways simultaneously requires careful consideration, particularly when factoring in the heterogeneity of AD pathology among patients, which complicates the creation of a universal multi‐target approach. This complexity highlights the need for precision in drug design to address the varied and interconnected aspects [160, 165, 175, 218]. Another major challenge of MTDD is drug delivery. It is very important to make sure MTDs reach within the brain while at the same time passing through the BBB [219]. Moreover, multiple targeting increases off‐target risk and might result in side effects that outweigh the benefits of treatment [220, 221].

Another major concern is the regulatory evaluation. The absence of standardized guidelines for assessing polypharmacological agents complicates their preclinical and clinical assessment, which makes it harder to justify efficacy and safety in a unified model [203]. Preclinical testing of multi‐target therapies presents additional difficulties, as existing animal models and testing frameworks often fail to accurately reflect the intricate interactions of multiple targets [222]. Also, understanding the pharmacokinetics (PK) and pharmacodynamics (PD) of this type of compound is crucial. Because MTDLs act on multiple sites, predicting their absorption, distribution, metabolism, excretion, and toxicity becomes more complex compared to single‐target drugs. However, CADD tools have shown the potential to improve these predictions. Another challenge is drug toxicity and safety. In cancer therapy, researchers found that combination therapies often result in accumulated side effects or unpredictable drug–drug interactions. Similarly, MTDLs may carry the risk of compound‐related toxicity due to the involvement of multiple pathways [223].

In addition, due to the high regulatory barriers for new multi‐target therapies, current approval processes are much better oriented for single‐target drugs and require adaptations that may slow the development and enhance the costs. The major barrier to the high‐scale adoption of this approach is the high cost of research and development coupled with the possible high expenses associated with bringing it to the market [224]. Other limitations of MTDD approaches include loss of potency at individual targets, failure to predict long‐term effects, and inability to optimize doses. There is also poor knowledge regarding the optimization of combinations of targets to avoid unwanted biological consequences. These limitations need careful consideration and extensive preclinical and clinical testing [202, 225, 226]. To unlock the full potential of MTDD in treating AD, further research and regulatory collaboration are needed to develop innovative solutions that address these issues effectively.

## 7 Conclusion and Future Directions

MTDD is transforming modern drug development, offering a remarkable avenue for the treatment of complex diseases. Today, MTDD is no longer just a theoretical concept; it has become a practical approach in real‐world drug discovery, particularly in cases where single‐target strategies have proven inadequate. The development of MTDD is not only essential for complex diseases like cancer, neurodegenerative diseases, and autoimmune diseases, but also creates a new path for future drug development strategies. The biological rationale for the development of multi‐targeting strategies is strong and validated by drugs like the memantine‐donepezil combinations. MTDD will be effective by addressing several challenges; for example, the deficiency of BBB permeability. Adaptation of these models from theory to the clinic will be critical for MTDD to realize its potential in the treatment of AD. However, these approaches have yet to see widespread adoption in pharmaceutical development. Emerging technologies such as AI/ML‐powered compound generation, PROTAC‐mediated degradation, and RNA‐based gene silencing further support these approaches by increasing design precision and translational potential. Further development of MTDD will require innovations in delivery systems, regulatory adaptation, and interdisciplinary collaboration. The future of MTDD in AD depends on improving drug delivery systems, particularly for crossing the BBB. An essential focus should be on personalized medicine, recognizing the diverse pathology of AD among individuals. The integration of computational biology, systems pharmacology, and clinical research will be essential in translating MTDD strategies from design to practice. To fully harness the promise of MTDD in Alzheimer's therapy, future work must prioritize the clinical translation of these strategies by addressing unresolved gaps in target validation, delivery system optimization, and regulatory readiness. Continued innovation in MTDD holds the potential to transform the treatment landscape for AD, offering new hope for millions of people affected by this debilitating disease.

## Author Contributions

**Md Saad Hossain:** conceptualization, formal analysis, investigation, methodology, visualization, writing – original draft. **Md Hamed Hussain:** formal analysis, project administration, supervision, writing – review and editing.

## Ethics Statement

This review did not involve any human or animal subjects and, therefore, does not require ethical approval.

## Conflicts of Interest

The authors declare no conflicts of interest.

## Data Availability

This is a review article and does not contain any original data. All data discussed are from previously published studies and are fully cited in the reference list.
